# Radiomics to predict outcomes and abscopal response of patients with cancer treated with immunotherapy combined with radiotherapy using a validated signature of CD8 cells

**DOI:** 10.1136/jitc-2020-001429

**Published:** 2020-11-13

**Authors:** Roger Sun, Nora Sundahl, Markus Hecht, Florian Putz, Andrea Lancia, Angela Rouyar, Marina Milic, Alexandre Carré, Enzo Battistella, Emilie Alvarez Andres, Stéphane Niyoteka, Edouard Romano, Guillaume Louvel, Jérôme Durand-Labrunie, Sophie Bockel, Rastilav Bahleda, Charlotte Robert, Celine Boutros, Maria Vakalopoulou, Nikos Paragios, Benjamin Frey, Jean-Charles Soria, Christophe Massard, Charles Ferté, Rainer Fietkau, Piet Ost, Udo Gaipl, Eric Deutsch

**Affiliations:** 1 Department of Radiation Oncology, Gustave Roussy, Villejuif, Île-de-France, France; 2 Institut Gustave Roussy, Inserm, Radiothérapie Moléculaire et Innovation Thérapeutique, Paris-Saclay University, Villejuif, Île-de-France, France; 3 Paris-Saclay University Faculty of Medicine, Le Kremlin-Bicetre, Île-de-France, France; 4 Department of Radiation Oncology, University Hospital Ghent, Gent, Oost-Vlaanderen, Belgium; 5 Department of Radiation Oncology, Friedrich-Alexander-Universität Erlangen-Nürnberg, Erlangen, Germany; 6 Department of Radiation Oncology, Fondazione IRCCS Policlinico San Matteo, Pavia, Lombardia, Italy; 7 TheraPanacea, Paris, France; 8 Drug Development Department, Gustave Roussy, Villejuif, Île-de-France, France; 9 Departement of Medicine, Gustave Roussy, Villejuif, Île-de-France, France; 10 CentraleSupélec, Gif-sur-Yvette, Île-de-France, France

**Keywords:** radioimmunotherapy, translational medical research, tumor biomarkers, tumor microenvironment

## Abstract

**Background:**

Combining radiotherapy (RT) with immuno-oncology (IO) therapy (IORT) may enhance IO-induced antitumor response. Quantitative imaging biomarkers can be used to provide prognosis, predict tumor response in a non-invasive fashion and improve patient selection for IORT. A biologically inspired CD8 T-cells-associated radiomics signature has been developed on previous cohorts. We evaluated here whether this CD8 radiomic signature is associated with lesion response, whether it may help to assess disease spatial heterogeneity for predicting outcomes of patients treated with IORT. We also evaluated differences between irradiated and non-irradiated lesions.

**Methods:**

Clinical data from patients with advanced solid tumors in six independent clinical studies of IORT were investigated. Immunotherapy consisted of 4 different drugs (antiprogrammed death-ligand 1 or anticytotoxic T-lymphocyte-associated protein 4 in monotherapy). Most patients received stereotactic RT to one lesion. Irradiated and non-irradiated lesions were delineated from baseline and the first evaluation CT scans. Radiomic features were extracted from contrast-enhanced CT images and the CD8 radiomics signature was applied. A responding lesion was defined by a decrease in lesion size of at least 30%. Dispersion metrices of the radiomics signature were estimated to evaluate the impact of tumor heterogeneity in patient’s response.

**Results:**

A total of 94 patients involving multiple lesions (100 irradiated and 189 non-irradiated lesions) were considered for a statistical interpretation. Lesions with high CD8 radiomics score at baseline were associated with significantly higher tumor response (area under the receiving operating characteristic curve (AUC)=0.63, p=0.0020). Entropy of the radiomics scores distribution on all lesions was shown to be associated with progression-free survival (HR=1.67, p=0.040), out-of-field abscopal response (AUC=0.70, p=0.014) and overall survival (HR=2.08, p=0.023), which remained significant in a multivariate analysis including clinical and biological variables.

**Conclusions:**

These results enhance the predictive value of the biologically inspired CD8 radiomics score and suggests that tumor heterogeneity should be systematically considered in patients treated with IORT. This CD8 radiomics signature may help select patients who are most likely to benefit from IORT.

## Introduction

The introduction of immune-checkpoint inhibitors has profoundly changed the immuno-oncology (IO) and the treatment of patients with metastatic cancers.^1 2^ Unfortunately, despite the use of patients stratification criteria, the average response rate (RR) remains low while being significantly volatile (20%–50%).^3^ Strong evidence indicates that radiotherapy (RT) can invoke both local and systemic immunostimulatory effects that could synergize with immunotherapy in systemic tumor control.^4–8^ However, open questions about modalities to improve IORT efficacy, such as the choice and number of lesions to irradiate,^9 10^ are still pending. Patient’s stratification becomes a necessity and the development of innovative biomarkers to optimize IORT paves the way to precision medicine in cancer care and improved clinical outcomes.

Quantitative imaging biomarkers are of great interest. Medical images are non-invasive, part of standard clinical protocol and reflect the whole tumor burden for which every single lesion can be analyzed, contrary to traditional biopsy.^11^ This is of crucial importance with IORT, where disease and treatment encompass and have to account for intrapatient heterogeneity. Lesions have different microenvironments and may receive either local and/or systemic treatment effects leading to heterogeneous patterns of global response.^12^

Evidence-based radiomic biomarkers have shown promising results to predict response to immunotherapy,^13–18^ but to the best of our knowledge, there is no evidence yet in IORT. Our team previously validated a biologically inspired radiomics-signature trained to estimate from conventional contrast-enhanced CT scans the abundance of CD8 T-cells and demonstrated correlations with outcomes of patients treated with immunotherapy.^13^ Here, we aimed to evaluate in a cohort of patients treated with IORT whether this tool can predict tumor response at a lesion-based level. We evaluated also whether this radiomics signature could help to assess disease spatial heterogeneity at a patient-level for clinical outcome prediction, and finally evaluated the impact of irradiation on the lesion radiomic scores.

## Methods

### Data and study design

Patients with metastatic solid tumors from three centers included in clinical trials of IORT were retrospectively screened for inclusion.

Patients for whom contrast-enhanced CT scans were available at both baseline and after start of treatment (first follow-up) were included for analysis.

Six clinical trials were considered for this study (SABR-PDL1: NCT02992912, MEL-IPI-RX: NCT01557114, ‘ghent-nivo’: NCT02821182,^19^ ‘ghent-ipi: NCT02406183,^20^ ‘ghent-pembro’: NCT02826564,^21^ ST-ICI: NCT03453892). Immunotherapy consisted of anticytotoxic T-lymphocyte-associated protein 4 (ipilimumab), antiprogrammed cell death protein 1 (anti-PD-1) (pembrolizumab, nivolumab) or antiprogrammed death-ligand 1 (anti-PD-L1) (atezolizumab) in monotherapy. The main RT regimen was stereotactic RT delivered in three fractions after the start of immunotherapy. Clinical, biological and imaging data at baseline and follow-up, and PD-L1 expression were retrieved when available.

The primary objective was to evaluate the association between the previously validated biologically inspired radiomics score of CD8 T-cells of a lesion and its response at the first follow-up CT.^13^ Secondary objectives were (1) to evaluate the association between the radiomics scores of the analyzed lesions (see Definition of analyzed lesions) and patients’ clinical outcomes at the first evaluation, progression-free survival (PFS) and overall survival (OS) and (2) to assess differences between irradiated and non-irradiated lesions. Potential clinical and biological confounding predictors of response (PFS, OS) and of abscopal response were analyzed.

### Image analysis

#### Definition of analyzed lesions

Analyzed lesions were any tumor (primary or secondary) that was identifiable on both baseline and follow-up CTs, with diameter ≥5 mm at baseline. Lesions that could not be accurately discriminated from surrounding tissues (ie, lung nodule adjacent or within atelectasis) or from other adjacent lesions at baseline or follow-up CTs (ie, confluent metastases) were not delineated and excluded. Irradiated and non-irradiated lesions were labeled.

#### Feature extraction and CD8 T-cells radiomics score computation

Radiomic features were extracted from contrast-enhanced CTs. All images had slice-thickness ≤5 mm and were reconstructed using soft or standard convolution kernels. Experienced physicians from one center (RS, AL) delineated lesions on baseline and follow-up scans. All the segmentations were reviewed by one physician (RS) to ensure a homogeneous segmentation method in accordance with the published signature. Two volumes of interest (VOIs) were segmented for each lesion: (1) the lesion itself and (2) a peripheral ring, created following the method published in the original study^13^ to compute the radiomics signature. The ring was created using automated dilatation and shrinkage of the tumor boundaries by 2 mm on each side, namely outside and inside the boundary, resulted in a ring with a thickness of 4 mm (online supplementary figure S1). Radiomic feature extraction was performed using LIFEx software V.3.44 (Local Image Feature Extraction, freeware, www.lifexsoft.org).^22^ Images were resized to 1×1×1 mm^3^ voxels using three-dimensional Lagrangian polygon interpolation. Hounsfield-units (HU) values in the VOIs were then resampled into 400 discrete values using absolute discretisation.^23^ The minimum and maximum bounds of the resampling interval were set to −1000 and 3000 HU, leading to a bin size of 10 HU.

10.1136/jitc-2020-001429.supp1 Supplementary data

**Figure 1:**
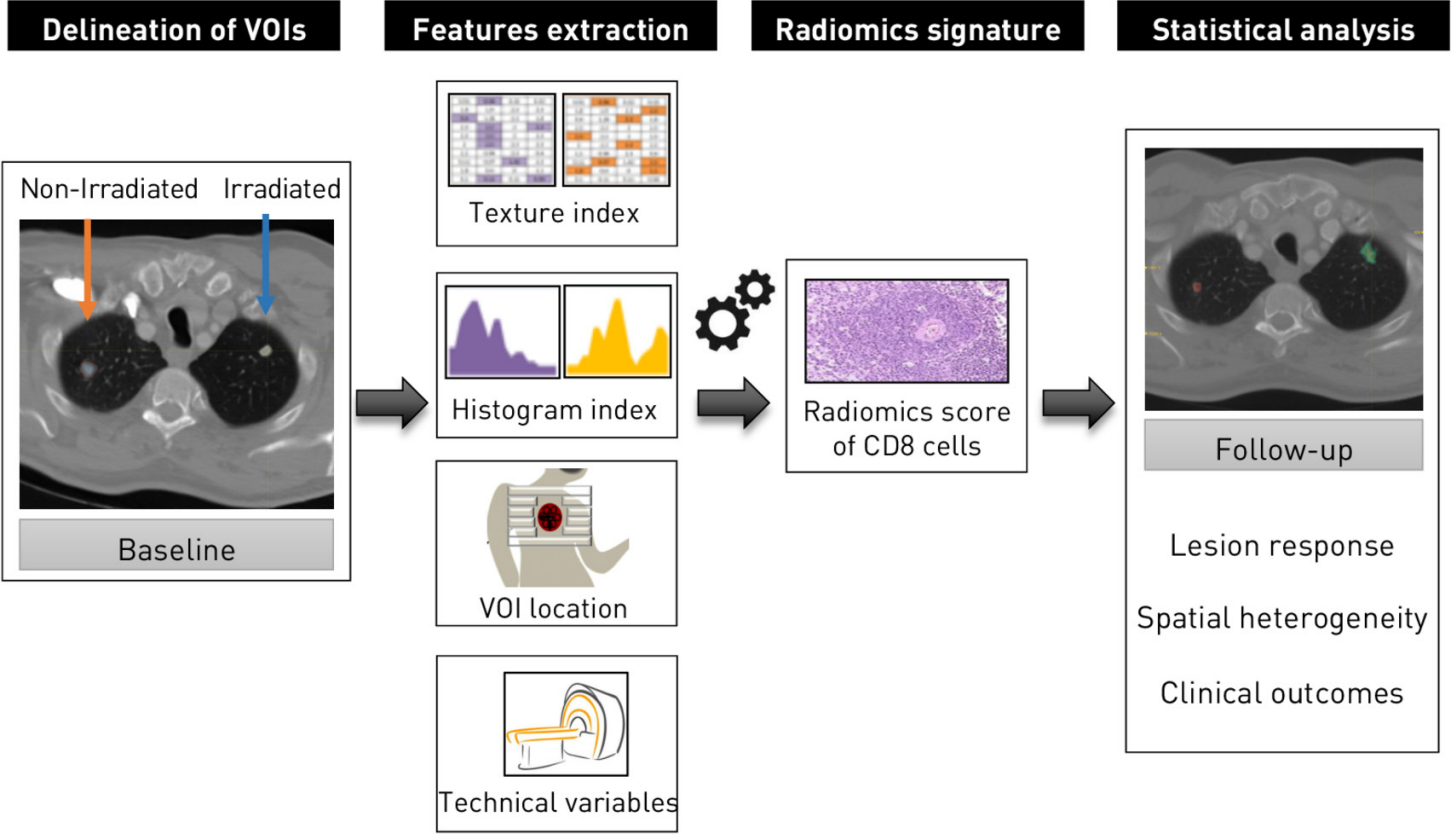
Radiomics workflow. Irradiated and non-irradiated lesions were delineated. After image preprocessing to discretise the voxel intensity and to resample the voxel size, features from the radiomics signature of CD8 T-cells published in Sun *et al*, *Lancet Oncol* 2018, were extracted, allowing the estimation of CD8 T-cells. Associations with outcomes were evaluated.

The previously validated radiomics score of CD8 T-cells consists of a linear interpolation on the basis of eight variables: five radiomic features extracted from each lesion, two discrete labels about lesion location and one imaging acquisition-related variable—the peak kilovoltage (kVp).^13^ The five features from the radiomics signature were extracted and normalized in the range 0–1. VOI location was labeled as adenopathy, head and neck, lung, liver or other (subcutaneous or abdominal lesions) and the kVp was retrieved from CT metadata. Coefficients from the validated signature were applied to these eight variables to obtain the radiomics score of each analyzed lesion (figure 1).

### Response evaluation

A lesion-wise evaluation of relative change in diameter between baseline and follow-up was carried out using RECIST V.1.1 criteria. A responding lesion was defined by a decrease in lesion size of >30%. A progressive lesion was defined by an increase in lesion size of >20%. To take into account the overall tumor burden and to assess the different patterns of response on a patient basis, mixed response was defined as the presence of both progressive and responding lesions, as opposed to patients presenting only responding (uniform response) or progressive lesions (uniform progression), irrespective of stable lesions or only stable lesions (stable disease (SD)). To compare patterns of response of non-irradiated lesions, occurrence of response in any non-irradiated lesion was defined as lesion-based abscopal response, while a decrease of >30% in the sum of the largest diameter of non-irradiated lesion was defined as a RECIST-based abscopal response.

### Spatial heterogeneity evaluation

To assess the impact of intrapatient interlesion CD8 radiomics score heterogeneity, minimum value, maximal value, mean value, SD and entropy of the lesion scores distribution were retrieved. For subgroup analysis of irradiated and non-irradiated lesions, when the number of lesions was >1, the minimum value was kept. Median value was used to separate patients into two groups. Patients with extreme values (<first quartile or >third quartile) regarding the minimum value of their lesions were also compared in a post hoc analysis.^24^ False discovery rate (FDR) was at 0.2 to correct for multiple comparisons.

### Statistical analysis

Comparisons between variables were performed using Wilcoxon signed-rank test or Kruskal-Wallis test for continuous variables, and Fisher’s exact test for categorical variables. Clustering of patients into two distinct groups was done on the basis of the median value, unless there was a validated cut-off (ie, biological variables). A threshold of <0.05 was defined for double-tailed p value’s significance. Statistical analyses were performed using R software V.3.6.0 (https://www.r-project.org/).^25^ OS and PFS were computed according to the Kaplan-Meier method and Cox proportional-hazards survival estimates. End points were death from any cause for OS, and any recurrence or death for PFS. Multivariate models included the clinically relevant variables that were significant in univariate analysis. No imputation was made for the missing data.

### Role of the funding sources

The funders of the study had no role in study design, data collection, data analysis, data interpretation or writing of the report. ED and RS had full access to all the data in the study and had final responsibility for the decision to submit for publication.

## Results

### Patient characteristics and analyzed lesions

Ninety-four patients were included from 1 July 2011 to 31 March 2019. Baseline characteristics of the patients are presented in table 1. All patients were diagnosed with advanced or metastatic solid tumors. Median age at baseline was 60.38 (IQR 50.68–69.04).

**Table 1:** Patient characteristics

	Level	Overall	GR	Erlangen	Ghent	P value
**n**		94	38	16	40	
**Age (median(IQR**))		60.38 (50.68 to 69.04)	57.38 (48.75 to 67.77)	64.04 (55.84 to 69.12)	62.80 (50.28 to 71.36)	0.320
**Sex (%**)	F	26 (27.7)	14 (36.8)	2 (12.5)	10 (25.0)	0.179
	M	68 (72.3)	24 (63.2)	14 (87.5)	30 (75.0)	
**Performance status (%**)	0	53 (56.4)	18 (47.4)	0 (0.0)	35 (87.5)	<0.001
	1	24 (25.5)	19 (50.0)	0 (0.0)	5 (12.5)	
	2	1 (1.1)	1 (2.6)	0 (0.0)	0 (0.0)	
	NA	16 (17.0)	0 (0.0)	16 (100.0)	0 (0.0)	
**Histology (%**)	CRC adenocarcinoma	21 (22.3)	21 (55.3)	0 (0.0)	0 (0.0)	NA
	HNSCC	5 (5.3)	0 (0.0)	5 (31.2)	0 (0.0)	
	Melanoma	39 (41.5)	14 (36.8)	0 (0.0)	25 (62.5)	
	NSCLC	14 (14.9)	3 (7.9)	11 (68.8)	0 (0.0)	
	TCC	13 (13.8)	0 (0.0)	0 (0.0)	13 (32.5)	
	Unknown primary	2 (2.1)	0 (0.0)	0 (0.0)	2 (5.0)	
**Number of prior lines of therapy (median(IQR**))		1.00 (0.00 to 1.00)	1.00 (1.00 to 2.00)	NA (NA, NA)	0.00 (0.00 to 1.00)	<0.001
**CT interval (median(IQR**))		85.50 (61.75 to 103.75)	63.00 (44.25 to 89.75)	128.00 (106.25 to 141.00)	83.50 (69.75 to 102.00)	<0.001
**N of VOI at baseline (median(IQR**))		3.00 (2.00 to 4.00)	3.00 (2.00 to 4.00)	3.00 (3.00 to 4.25)	3.00 (2.00 to 3.00)	0.089
**N of VOI of irradiated lesions at baseline (median(IQR**))		1.00 (1.00 to 1.00)	1.00 (1.00 to 1.00)	1.00 (1.00 to 2.00)	1.00 (1.00 to 1.00)	0.012
**N of VOI of non-irradiated lesions at baseline (median(IQR**))		2.00 (1.00 to 3.00)	2.00 (1.00 to 3.00)	2.50 (1.00 to 3.00)	2.00 (1.00 to 2.00)	0.583
**N of organs involved (%)**	< or =2	56 (59.6)	20 (52.6)	3 (18.8)	33 (82.5)	<0.001
	> or =3	38 (40.4)	18 (47.4)	13 (81.2)	7 (17.5)	
**IO therapy (%)**	Atezolizumab		24 (25.5)	24 (63.2)	0 (0.0)	0 (0.0)
	Ipilimumab		25 (26.6)	14 (36.8)	0 (0.0)	11 (27.5)
	Nivolumab		29 (30.9)	0 (0.0)	13 (81.2)	16 (40.0)
	Pembro		16 (17.0)	0 (0.0)	3 (18.8)	13 (32.5)
**N of IO cycles (median(IQR**))			4.00 (3.00 to 10.00)	4.00 (3.00 to 6.00)	3.00 (0.00 to 12.00)	5.50 (4.00 to 16.00)
**Time between RT and IO therapy (%**)	< or =14 days		35 (37.2)	0 (0.0)	14 (87.5)	21 (52.5)
	> day 14		59 (62.8)	38 (100.0)	2 (12.5)	19 (47.5)
**Hypofractionated RT (%**)	0		3 (3.2)	0 (0.0)	3 (18.8)	0 (0.0)
	1		91 (96.8)	38 (100.0)	13 (81.2)	40 (100.0)
**Stereotactic RT (%**)	0		7 (7.4)	0 (0.0)	7 (43.8)	0 (0.0)
	1		87 (92.6)	38 (100.0)	9 (56.2)	40 (100.0)
**RT: N of fractions (median(IQR**))			3.00 (3.00 to 3.00)	3.00 (3.00 to 3.00)	12.00 (10.75 to 15.00)	3.00 (3.00 to 3.00)
**RT: dose per fraction (median(IQR**))			8.00 (6.00 to 12.00)	13.00 (5.00 to 15.00)	3.50 (3.00 to 6.00)	8.00 (8.00 to 8.00)
**RT: total dose in EQD2 (median(IQR**))			36.00 (36.00 to 69.00)	75.00 (18.75 to 93.75)	51.55 (41.25 to 70.00)	36.00 (36.00 to 36.00)
**Follow-up (median(IQR**))			451.50 (256.50 to 633.75)	360.50 (243.00 to 624.75)	277.50 (187.50 to 394.25)	540.00 (376.00–817.75)
**PD-L1 (tumor cells) (median(IQR**))			5.00 (0.00–33.75)	NA (NA, NA)	30.00 (12.50–80.00)	1.00 (0.00–10.00)
**PD-L1 (immune cells) (median(IQR**))			5.00 (0.00–12.50)	NA (NA, NA)	8.75 (5.00–17.50)	1.00 (0.00–10.00)
**Baseline absolute neutrophil count (%**)		< or =7.5 G/L	68 (72.3)	27 (71.1)	5 (31.2)	36 (90.0)
		>7.5 G/L	17 (18.1)	11 (28.9)	2 (12.5)	4 (10.0)
		NA	9 (9.6)	0 (0.0)	9 (56.2)	0 (0.0)
**Baseline absolute lymphocyte count (%**)		> or =1 G/L	64 (68.1)	30 (78.9)	1 (6.2)	33 (82.5)
		<1 G/L	21 (22.3)	8 (21.1)	6 (37.5)	7 (17.5)
		NA	9 (9.6)	0 (0.0)	9 (56.2)	0 (0.0)
**Baseline neutrophil-to-lymphocyte ratio (%**)		< or =6	65 (69.1)	29 (76.3)	2 (12.5)	34 (85.0)
		>6	20 (21.3)	9 (23.7)	5 (31.2)	6 (15.0)
		NA	9 (9.6)	0 (0.0)	9 (56.2)	0 (0.0)
**Baseline LDH (%**)		< or =250	56 (59.6)	21 (55.3)	6 (37.5)	29 (72.5)
		>250	30 (31.9)	17 (44.7)	2 (12.5)	11 (27.5)
		NA	8 (8.5)	0 (0.0)	8 (50.0)	0 (0.0)
**Baseline albumin (%**)		> or =35	58 (61.7)	32 (84.2)	11 (68.8)	15 (37.5)
		<35	6 (6.4)	5 (13.2)	1 (6.2)	0 (0.0)
		NA	30 (31.9)	1 (2.6)	4 (25.0)	25 (62.5)
**Baseline CRP (%**)		< or =10 mg/L	58 (61.7)	18 (47.4)	9 (56.2)	31 (77.5)
		>10 mg/L	32 (34.0)	20 (52.6)	7 (43.8)	5 (12.5)
		NA	4 (4.3)	0 (0.0)	0 (0.0)	4 (10.0)
**Baseline RMH score (%**)		0	23 (24.5)	10 (26.3)	0 (0.0)	13 (32.5)
		1	25 (26.6)	18 (47.4)	5 (31.2)	2 (5.0)
		2	7 (7.4)	6 (15.8)	1 (6.2)	0 (0.0)
		3	3 (3.2)	3 (7.9)	0 (0.0)	0 (0.0)
		NA	36 (38.3)	1 (2.6)	10 (62.5)	25 (62.5)
**Baseline GR immune score (%**)		Low	26 (27.7)	16 (42.1)	0 (0.0)	10 (25.0)
		High	29 (30.9)	21 (55.3)	3 (18.8)	5 (12.5)
		NA	39 (41.5)	1 (2.6)	13 (81.2)	25 (62.5)

CRC, colorectal cancer; CRP, C reactive protein; EQD2, equivalent dose in 2 Gy fraction; GR, Gustave Roussy; HNSCC, squamous cell cancers of the head and neck; IO, immuno-oncology; LDH, lactate dehydrogenase; NA, not available; NSCLC, non-small cell lung cancer; PD-L1, programmed death-ligand 1; RMH, Royal Marsden Hospital score; RT, radiotherapy; TCC, Transitional cell carcinoma; VOI, volume of interest.

Two patients (2.1%) with irradiated brain metastases had no contrast-enhanced brain CTs available for imaging analysis of the irradiated lesion but were kept for the analysis of non-irradiated lesions response. Conversely, three patients (3.2%) had no identifiable non-irradiated analyzed lesion but were considered for the irradiated lesion response. Overall, 100 irradiated lesions and 189 non-irradiated lesions were analyzed at baseline and follow-up, leading to a total of 578 lesions. At baseline, median size of irradiated lesions was larger than non-irradiated lesions (26.9 mm, IQR (17.6–37.7) vs 21.5 mm, IQR (15.2–31.9), respectively, difference in location=3.6, 95% CI (0.3 to 6.8), p=0.034) (table 2).

**Table 2:** Characteristics of lesions analyzed at baseline

	Level	Irradiated	Non-irradiated	P value
n		100	189	
Location (%)	Node	38 (38.0)	64 (33.9)	0.015
	Brain	1 (1.0)	0 (0.0)	
	Liver	14 (14.0)	42 (22.2)	
	Head and neck	1 (1.0)	0 (0.0)	
	Bone	4 (4.0)	0 (0.0)	
	Other	17 (17.0)	42 (22.2)	
	Lung	25 (25.0)	39 (20.6)	
	Adrenal gland	0 (0.0)	2 (1.1)	
Lesion size (median (IQR))		26.92 (17.55 to 37.71)	21.54 (15.23 to 31.89)	0.034
Lesion volume (median (IQR))		4.84 (1.26 to 15.74)	2.83 (0.99 to 9.26)	0.093
CD8 radiomics score (median (IQR))		1.75 (1.55 to 1.97)	1.74 (1.54 to 1.97)	0.756

### Treatment

Nearly all patients underwent hypofractionated RT (n=91 of 94; 96.9%). Six patients had two irradiated metastases (6.4%), and one had three irradiated metastases (1.1%). Median number of fractions and dose by fraction given were three fractions (IQR (3–3)) of 8 Gy (IQR (6–12)). Median number of IO therapy cycles given was 4 (IQR (3–10)). Median time between the start of IO therapy and the start of RT was 21 days (IQR (8–24)). Sixteen patients started RT before IO therapy (median interval: −5.5, IQR (−7–−1.75)). Median follow-up was 14.8 months (IQR (8.4–20.8)). Median time between baseline and follow-up CT was 2.8 months (IQR (2.0–3.4)).

### Patient outcomes description

#### Lesion response

The median per cent change in lesion diameter was −13.0% (IQR (−28.9–+10.1)) for irradiated metastases versus +11.2% (IQR (−11.0–+40.5)) for non-irradiated metastases (p<0.0001), with respectively 16% progressive lesions (n=16 of 100 lesions) and 23% responding lesions (n=23 of 100) for irradiated metastases and 38% progressive lesions (n=72 of 189) and 17% responding lesions (n=32 of 189) for non-irradiated metastases.

#### Patient response

Regarding patient response at the first follow-up CT, 6 complete responses, 22 partial responses, 24 SD and 42 progressive disease were observed according to RECIST V.1.1, yielding an objective RR of 29.9% (28 of 94 patients). More precisely, 24.5% (n=23) showed uniform response, 38.3% (n=36) showed uniform progression, 12.8% (n=12) showed mixed response and 24.5% (n=23) presented only stable lesions.

**Figure 2:**
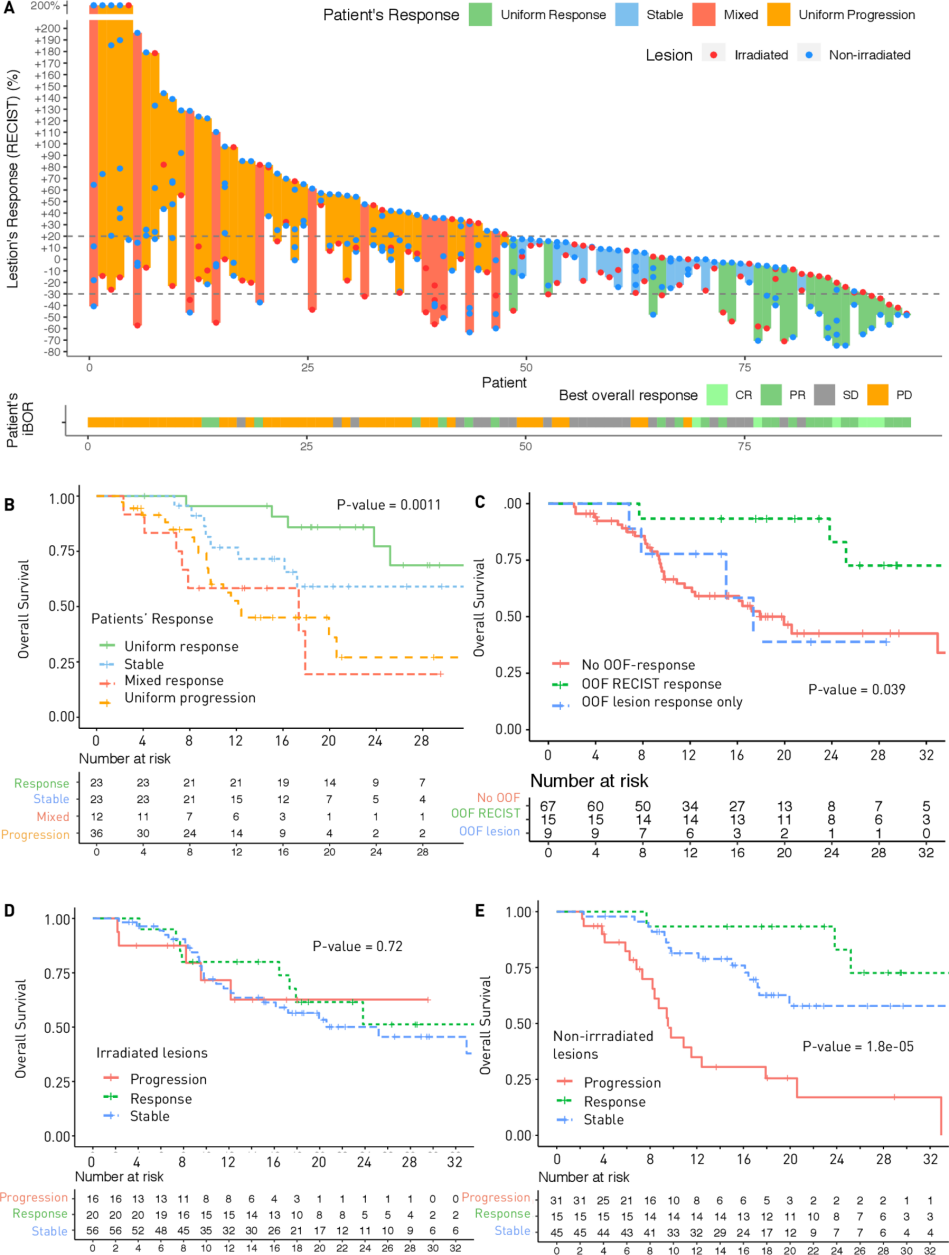
(A) Response kinetics curve depicting individual lesion responses (red dots corresponding to irradiated lesions, blue dots corresponding to non-irradiated lesions) on a patient-to-patient basis. (B) Kaplan-Meier curves of overall survival according to patients’ patterns of response. (C) Kaplan-Meier curves of overall survival according to out-of-field response (OOF). Out-of-field response which fulfilled RECIST V.1.1 criteria (30% decrease in the sum of the aggregate diameters of the non-irradiated lesions) showed higher overall survival (OS) than the rest of the population. (D, E) Kaplan-Meier curves of overall survival according to aggregate diameter changes of irradiated lesions (D) or non-irradiated lesions (E).

Median OS was 25.2 months (95% CI (17.3–NA)) and median PFS was 4.7 months (95% CI (3.5–6.7)) (online supplementary figure S2). Patients with uniform responses had significantly higher OS (HR=0.20, 95% CI (0.08 to 0.53), p=0.0011), while OS of patients with mixed response was not significantly different from that of patients with uniform progression (p=0.84) (figure 2A).

#### Out-of-field response

**Figure 3A:**
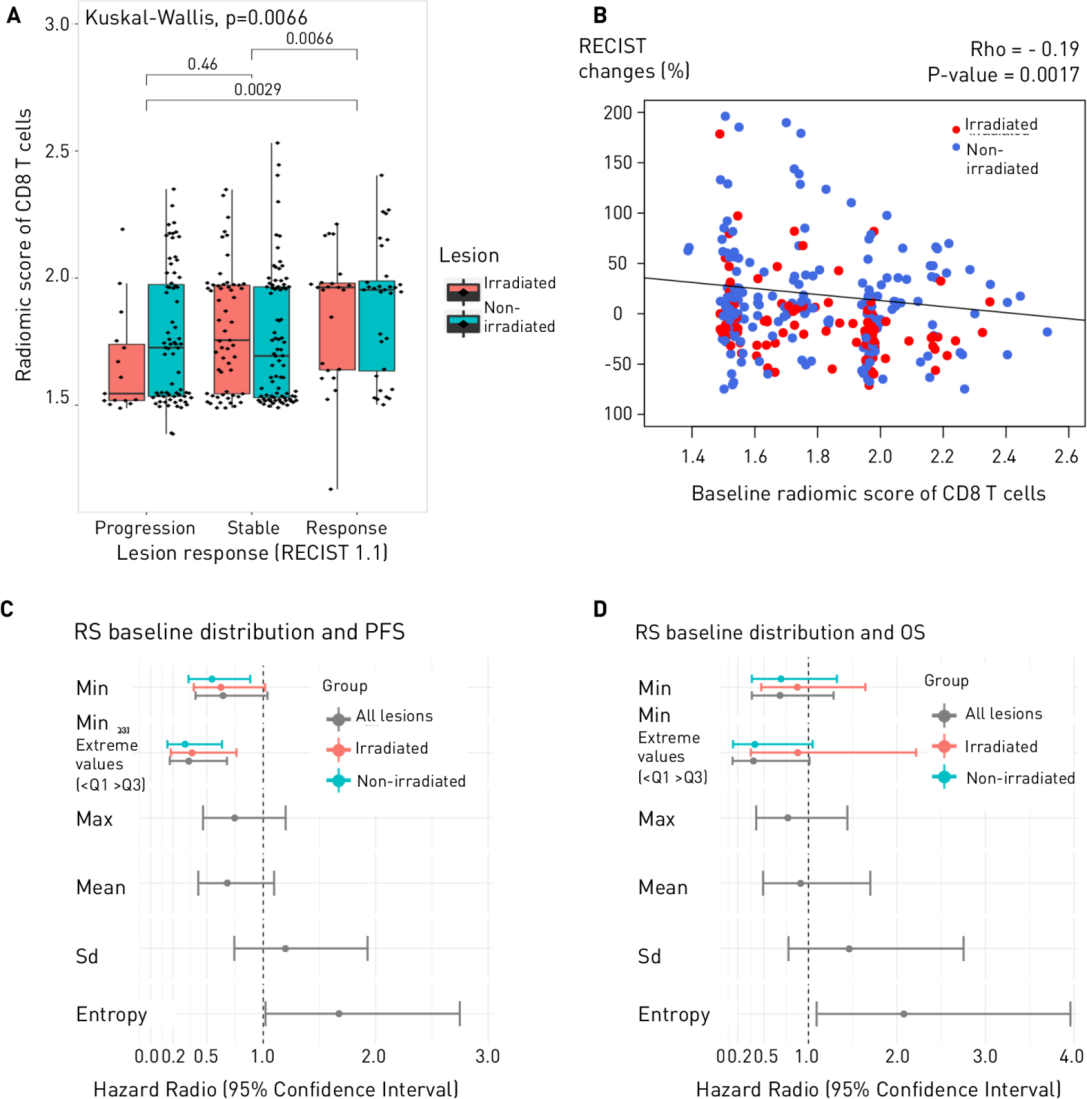
Lesion response according to baseline radiomics score. Responding lesions corresponded to lesions with a decrease in tumor size >30% (partial response and complete response). (B) Scatter plot of changes in tumor size (%) according to baseline radiomics score. (C, D) Associations between distribution metrices of the radiomics score of CD8 T-cells and clinical outcomes (progression-free survival (PFS), overall survival (OS)).

For the 91 patients with non-irradiated lesions available for analysis, out-of-field response using aggregate diameter of non-irradiated lesions according to RECIST (RECIST-based abscopal response) at the first follow-up CT was 16.5% (n=15). Using response defined by 30% reduction in any single non-irradiated lesions (lesion-based abscopal response), the non-irradiated RR was 26.4% (n=24). While both definitions were significantly associated with OS (p=0.011 and p=0.041, respectively), only patients with RECIST-based abscopal response had significantly higher OS than those with no out-of-field response (HR=0.31, 95% CI (0.11 to 0.88), p=0.027), contrary to the six patients with lesion-based abscopal response who did not achieve the criteria for RECIST-based abscopal response (p=0.89) (figure 2C). For irradiated lesions, diameter changes according to RECIST were not associated with OS (p=0.72) (figures 2D–E and 3).

### CD8 radiomics score and association with patient outcomes

At baseline, median value of the CD8 radiomics score was not significantly different between the two groups of lesions (irradiated and non-irradiated) (1.75, IQR (1.55–1.97) vs 1.74, IQR (1.54–1.97), respectively, difference in location=0.005, 95% CI (−0.03 to 0.04), p=0.76). Radiomics score of CD8 T-cells was not correlated with lesion size (Spearman’s rho=−0.005, p=0.93).

#### Lesion response

Association between the CD8 radiomics score of a lesion at baseline and its response according to RECIST was significant (p=0.0066) with responding lesion having a higher CD8 radiomics score (median (IQR)=1.95 (1.64–1.98) and 1.72 (1.53–1.97) for stable or progressive lesions (area under the receiving operating characteristic curve (AUC)=0.63, 95% CI (0.56 to 0.71), p=0.0020) (figure 3A–B).

#### Patient response

Several metrices were evaluated to decipher whether heterogeneity of the CD8 score at baseline was associated with clinical outcomes (figure 3C–D and online supplementary table S1).

Patients with uniform progression had lower CD8 radiomics scores at baseline (based on the minimal value across their lesions) than those with uniform response (p=0.012) and mixed response (p=0.0073), but patients with either mixed response or uniform progression had more heterogeneous CD8 radiomics scores across their lesions (higher entropy of the distribution of CD8 T-cells radiomics score) than patients with SD or uniform response (online supplementary figure S4).

Entropy of the distribution of the CD8 radiomics scores, was associated with PFS, patients with higher value than the median having poorer PFS (HR=1.67, 95% CI (1.02 to 2.75), p=0.040, FDR=0.080), and remained significantly associated with OS (HR=2.08, 95% CI (1.09 to 3.96), p=0.023, FDR=0.17).

Minimal value, maximal value, mean and SD of the lesion scores were not significantly associated with PFS or OS. However, a statistical trend was identified between PFS and the minimal value of the CD8 radiomics score across all lesions (p=0.07), and significance was reached when looking only at patients with extreme values (HR=0.34, 95% CI (0.17 to 0.68), p=0.0021) (online supplementary table S1).

CD8 radiomics scores on the follow-up CT were not significantly different from baseline CT.

#### Irradiated and non-irradiated lesions subgroup analysis

On a lesion level, for both irradiated and non-irradiated lesions, the radiomics scores of responding lesions were higher than those of progressive lesions (AUC=0.75, 95% CI (0.58 to 0.92), p=0.0090 and AUC=0.75, 95% CI (0.51 to 0.74), p=0.040, respectively). There was no difference in tumor size at baseline between responding and progressive lesions (online supplementary figure S5).

On a patient level, minimum value of the CD8 radiomics score across non-irradiated lesions was significantly associated with PFS (HR=0.37, 95% CI (0.18 to 0.76), p=0.0067, FDR=0.022). For the irradiated lesions, this value was not significantly associated with PFS (HR=0.62, 95% CI (0.38 to 1.02), p=0.059, FDR=0.10). When looking at the extreme values, the minimum values of both non-irradiated and irradiated lesions were associated with PFS (p=0.0011 and p=0.0067, respectively).

Regarding out-of-field response, heterogeneous CD8 radiomics scores based on entropy of the distribution were associated with RECIST-based abscopal response (AUC=0.70, 95% CI (0.56 to 0.84), p=0.014).

#### Clinical and biological predictors of outcomes and multivariate analysis

Univariate analysis of OS and PFS are summarized in supplemental data (online supplementary table S2, S3). A performance status (PS) >0, an interval between IO therapy and RT start >14 days, high level of lactate dehydrogenase (>250 UI/L), high level of C reactive protein (CRP) (>10 mg/L) and high entropy of the CD8 T-cells radiomics scores distribution were significantly associated with both poorer OS and PFS. In the multivariate analysis of OS including these factors, only CRP (HR=3.34; 95% CI (1.48 to 7.52), p=0.0036) and the entropy of the CD8 T-cells radiomics scores distribution remained significant (HR=2.64; 95% CI (1.21 to 5.75), p=0.015) (online supplementary table S4). For PFS, only the PS remained significant in multivariate analysis (HR=4.6; 95% CI (2.28 to 9.28), p<0.0001) (online supplementary table S4). Center of inclusion was not a significant confounding factor when included in the multivariate analyses (online supplementary table S5). In this cohort, we did not identify a prognostic role of PD-L1 either for PFS or OS (online supplementary table S2, S3). Royal Marsden Hospital score and Gustave Roussy Immune Score were associated with PFS, but not with OS.^14 26–29^

## Discussion

IORT is becoming widely used to potentiate immune response. Imaging biomarkers would be very valuable as they could assess the ensemble of the disease extent and its spatial heterogeneity, and may help in the choice of the target lesion(s) to irradiate in order to optimize synergistic effects.^10 30^ Some authors hypothesized that interlesion heterogeneity of bulky disease may limit the probability of RT-induced systemic antitumor immune response, especially in single-site abscopal approach.^9^ Moreover, some studies suggested that the location of irradiation may impact how the immune response is elicited, but there is still no evidence regarding the optimal strategy.^31^

In this study, we demonstrated that the predictive value of a radiomics score of CD8 T-cells previously validated in a cohort of patients treated with IO monotherapy could still be informative in a context of radio-immunotherapy combination.^13^ Indeed, lesions with high radiomics score at baseline were associated with decrease in the lesion size at the follow-up CT, irrespective of irradiation. Moreover, our results suggest that this tool could also be used to assess tumor heterogeneity, which was associated with patient outcome. On a per-patient basis, the lowest radiomics score of the patient’s non-irradiated lesions was associated with PFS. This index was concordant with the observation that outcomes of non-irradiated lesions (vs irradiated lesion) were more predictive of the patient outcome (figures 2D–E and online supplementary figure S4). Therefore, this radiomics score of CD8 T-cells could be used to identify the lesions with the lowest CD8 tumor infiltration. Such lesions could benefit from local treatment since irradiation was associated with more responding lesions. On the contrary, the non-irradiated lesions with the highest radiomics scores would be more likely to present out-of-field response to IO therapy. Indeed, association with PFS and OS was even more important when considering patients with extreme values (although not significant for OS in this study). Entropy of the distribution of the radiomics scores of CD8 T-cells was associated with both PFS and OS and could discriminate patients who will present uniform response from those with uniform PD and mixed response. A high entropy indicated heterogeneous scores of CD8 infiltration and was associated with poorer outcomes. This index highlighted the impact of tumor heterogeneity on the prognosis of the patient and the importance of assessing the whole disease instead of only one lesion.

These results confirm the potential of radiomics to assess spatial heterogeneity as a non-invasive ‘multiple virtual biopsies’.^14^ Korpics *et al* recently presented similar preliminary results in a population of patients treated with IORT, adding further external validation, although they only evaluated irradiated lesions.^24^ They analyzed 68 patients and 139 irradiated tumors and showed associations between the CD8 radiomics score and local tumor response (OR=10.21, 95% CI (1.76 to 59.17); p=0.010), OS (HR=0.39, 95% CI (0.20 to 0.75); p=0.005) and PFS (HR=0.47, 95% CI (0.26 to 0.85); p=0.013), using a 25% percentile cut-off for dichotomization of patients.

To the best of our knowledge, this previously published radiomics signature of CD8 T-cells predicting response to IO is the first to be validated by several subsequent independent studies.^13 24^ We acknowledge however that this study had some limitations. The magnitude of the association between the CD8 radiomics score and the lesion response, although significant, was limited with overlaps between the different groups of response. However, considering that the radiomic signature has not been retrained in this cohort, it was interesting to note that results seemed concordant with those published in the original study (radiomics score between patients with Complete Response or Partial Response (CRPR) and Stable Disease or Progressive Disease (SDPD) at 3 months ‘Wilcoxon’s difference in location 0.12, 95% CI 9.9×10^−^⁶ to 2.3; p=0.049’, corresponding to an AUC of 0.62 in the immunotherapy cohort).^13^ Therefore, these results comfort further the reliability of the validated signature. Given the relatively low number of patients and the number of lesions evaluated per patient, we acknowledge that the analysis of the spatial heterogeneity should be interpreted with caution and should be only considered as exploratory. Overall, validation of these data still needs to be done in prospective and, if possible, randomized trials with homogeneous patients to refine and improve the performance of the signature in specific indications. Moreover, the association between the IORT protocol and the primitive tumor type inherent to the different clinical trials may have brought potential confounding factors limiting the analysis of the predictive factors. Still, this study addressed several pending questions regarding IORT. Tumor heterogeneity has shown to be important and correlates to certain extent with outcomes. However, the ‘abscopal’ out-of-field response should not be based only on the response of one non-irradiated lesion, as frequently reported.^32^ OS of patients who do not fulfill out-of-field RECIST criteria may not be different from patients without out-of-field response. Rather, we should stick with RECIST V.1.1. criteria which recommend not to use irradiated lesions as RECIST-target lesions to assess treatment response, and analyze several non-irradiated RECIST-target lesions to have information about the whole disease and the response heterogeneity.^19 33 34^ Regarding RT modalities, an interval between RT and IO therapy of <2 weeks was associated with better PFS and OS, although not significant in multivariate analysis. Interestingly, some variables such as liver metastasis, or scores like the Royal Marsden Hospital score and the Gustave Roussy Immune Score known to be prognostic factors with IO therapy,^14 26–29^ were only associated with PFS and not with OS in this cohort of patients treated with IORT. This may reflect potential long-lasting RT-induced immune response for which new biomarkers specifically validated for IORT strategies should be identified.

## Conclusion

Our findings confirm the predictive value of the previously published CD8 radiomics score in patients with solid tumors treated with IORT combinations.^13^ Moreover, the results suggest that global spatial reasoning on this signature through a statistical analysis of the distribution of this score across patient’s lesions could be a promising method to assess tumor heterogeneity and to predict clinical outcomes. Overall, this study asserts the potential of radiomics to help clinicians characterize the whole disease and to move toward in precision medicine.
