# Supplementary material for: Radiomics to predict outcomes and abscopal response of patients with cancer treated with immunotherapy combined with radiotherapy using a validated signature of CD8 cells
Source: J Immunother Cancer. 2020 Nov 13;8(2):e001429. doi: 10.1136/jitc-2020-001429 (PMC7668366; doi:10.1136/jitc-2020-001429)
Supplement: Supplementary data [file jitc-2020-001429supp001.pdf]

Supplementary Tables

Table S1. Association of CD8 radiomics score and clinical outcomes

PFS and CD8 radiomics score Baseline values		Variable Tested	n	n event	Hazard Ratio	lower CI	upper CI	P-value	FDR
All lesions	Min	x>1.56	94	69	0.64	0.40	1.04	0.07*	0.10
	**Min (extreme values)**	**xhigh**	**48**	**35**	**0.34**	**0.17**	**0.68**	**0.0021*****	**0.011**
	Max	x>1.96	94	69	0.75	0.46	1.20	0.23	0.26
	Mean	x>1.75	94	69	0.68	0.42	1.10	0.11	0.14
	Standard deviation	x>0.1	93	68	1.20	0.74	1.93	0.46	0.46
	**Entropy**	**x>1.09**	**87**	**65**	**1.67**	**1.02**	**2.75**	**0.04****	**0.080**
Irradiated	Min	x>1.73	88	65	0.62	0.38	1.02	0.059*	0.10
	**Min (extreme values)**	**xhigh**	**44**	**32**	**0.37**	**0.18**	**0.76**	**0.0067*****	**0.022**
Non-irradiated	**Min**	**x>1.61**	**91**	**68**	**0.54**	**0.34**	**0.88**	**0.014****	**0.035**
	**Min (extreme values)**	**xhigh**	**46**	**33**	**0.31**	**0.15**	**0.63**	**0.0011*****	**0.011**

OS and CD8 radiomics score Baseline values		Variable Tested	n	n event	Hazard Ratio	lower CI	upper CI	P-value	FDR
All lesions	Min	x>1.56	94	40	0.68	0.36	1.28	0.23	0.43
	**Min (extreme values)**	**xhigh**	**48**	**18**	**0.38**	**0.14**	**1.01**	**0.045****	**0.17**
	Max	x>1.96	94	40	0.77	0.41	1.44	0.41	0.59
	Mean	x>1.75	94	40	0.91	0.49	1.7	0.77	0.78
	Standard deviation	x>0.1	93	39	1.46	0.77	2.75	0.24	0.43
	**Entropy**	**x>1.09**	**87**	**40**	**2.08**	**1.09**	**3.96**	**0.023****	**0.17**
Irradiated	Min	x>1.73	88	39	0.87	0.47	1.64	0.68	0.78
	Min (extreme values)	xhigh	44	18	0.88	0.35	2.22	0.78	0.78
Non-irradiated	Min	x>1.61	91	39	0.69	0.36	1.32	0.26	0.43
	Min (extreme values)	xhigh	46	19	0.39	0.15	1.05	0.052*	0.17

Table S2 OS univariate analysis

	Variable tested	n	n events	Hazard Ratio [95% CI]	p
Cohort	Erlangen	94	40	0.57 [0.17; 1.95]	0.37
(ref=Gustave Roussy)	Ghent	94	40	0.64 [0.33; 1.22]	0.17
Age	>60.38	94	40	1.15 [0.62; 2.15]	0.66
Sex	M	94	40	0.85 [0.44; 1.64]	0.63
**Performance status**	**PS 1-2**	**78**	**37**	**2.65 [1.33; 5.29]**	**0.0082**
Histology	HNSCC	94	40	2 [0.43; 9.38]	0.38
(ref=colorectal cancer)	melanoma	94	40	0.64 [0.28; 1.44]	0.28
	NSCLC	94	40	0.15 [0.02; 1.16]	0.069
	TCC	94	40	1.12 [0.43; 2.92]	0.81
	unknown primary	94	40	0.49 [0.06; 3.92]	0.50
N of previous lines	>1	54	28	1.17 [0.44; 3.12]	0.76
N of IO cycles	>4	93	40	0.54 [0.28; 1.04]	0.059
**IO-RT interval**	**RT >d14 after IO**	**94**	**40**	**2.67 [1.18; 6.07]**	**0.010**
RT n of fractions	>3	94	40	0.48 [0.12; 2.03]	0.27
RT dose per fraction	>8	94	40	1.54 [0.82; 2.91]	0.19
RT total dose EQD2	>36	94	40	1.12 [0.59; 2.12]	0.72
N of organs involved	>or= 3	94	40	1.14 [0.6; 2.18]	0.69
Involved: Node	Yes	94	40	1.51 [0.74; 3.09]	0.24
Involved: Brain	Yes	94	40	1.56 [0.37; 6.51]	0.57
Involved: Liver	Yes	94	40	1.6 [0.83; 3.09]	0.17
Involved: Head&neck	Yes	94	40	8.09 [1.87; 35.12]	0.031
Involved: Bone	Yes	94	40	0.53 [0.13; 2.22]	0.34
Involved: Sub cutaneous	Yes	94	40	0.63 [0.31; 1.3]	0.20
Involved: Other	Yes	94	40	2.11 [1.11; 3.99]	0.027
Involved: Lung	Yes	94	40	1.19 [0.63; 2.23]	0.59
Involved: Adrenal gland	Yes	94	40	0.94 [0.13; 6.85]	0.95
Irradiated: node	Yes	94	40	1.3 [0.69; 2.44]	0.42
Irradiated: Brain	Yes	94	40	1.9 [0.26; 14.02]	0.57
Irradiated: Liver	Yes	94	40	1.15 [0.45; 2.96]	0.77
Irradiated: Head&neck	Yes	94	40	57 [5.04; 644.6]	0.017
Irradiated: Bone	Yes	94	40	NC	0.13
Irradiated: Sub cutaneous	Yes	94	40	0.85 [0.39; 1.86]	0.68
Irradiated: Other	Yes	94	40	2.13 [0.29; 15.69]	0.51
Irradiated: Lung	Yes	94	40	0.71 [0.33; 1.55]	0.38
PD-L1 (tumor cells)	>5%	40	10	0.84 [0.24; 3]	0.79
PD-L1 (immune cells)	>5%	39	10	1.11 [0.31; 3.93]	0.87
Absolute neutrophil count	>7.5 G/l	85	39	2.36 [1.15; 4.84]	0.028
Absolute lymphocyte count	<1 G/l	85	39	2.26 [1.11; 4.62]	0.035
**LDH**	**>250**	**86**	**39**	2.48 [1.3; 4.73]	**0.0074**
Albumin	<35	64	30	2.85 [1.08; 7.52]	0.060
**CRP**	**>10 mg/l**	**90**	**38**	**2.74 [1.4; 5.35]**	**0.0039**
NLR	>6	85	39	1.2 [0.55; 2.62]	0.66
RMH	RMH > 0	58	29	1.55 [0.73; 3.32]	0.25
GRim	High	55	28	1.47 [0.69; 3.12]	0.32
**RS CD8 T cells: entropy across all lesions**	**>1.09**	**87**	**40**	**2.08 [1.09; 3.96]**	**0.023**
RS CD8 T cells: minimal value of non-irradiated lesions	>1.61	91	39	0.69 [0.36; 1.32]	0.26
	High (>Q3)	46	19	0.39 [0.15; 1.05]	0.052

IO: immuno-oncology; RT: radiotherapy; EQD2: equivalent dose in 2-Gy fraction; LDH: lactate dehydrogenase; CRP: C-reactive protein; NLR: neutrophil to lymphocyte ratio; RMH: Royal Marsden Hospital score; Grim: Gustave Roussy immune-score; RS: radiomics score.

Table S3 PFS univariate analysis

	Variable tested	n	n events	Hazard Ratio [95% CI]	p
Cohort	Erlangen	94	69	0.47 [0.22; 0.98]	0.044
(ref=Gustave Roussy)	Ghent	94	69	0.55 [0.33; 0.91]	0.021
Age	>60.38	94	69	0.95 [0.6; 1.53]	0.85
Sex	M	94	69	0.84 [0.5; 1.42]	0.52
**Performance status**	**PS 1-2**	**78**	**60**	5.66 [3.18; 10.08]	**1.90E-08**
Histology	HNSCC	94	69	0.91 [0.3; 2.72]	0.86
(ref=colorectal cancer)	melanoma	94	69	0.39 [0.21; 0.71]	0.002
	NSCLC	94	69	0.3 [0.13; 0.7]	0.0052
	TCC	94	69	0.45 [0.21; 0.97]	0.042
	unknown primary	94	69	0.26 [0.04; 1.99]	0.20
N of previous lines	>1	54	39	0.91 [0.38; 2.17]	0.82
N of IO cycles	>4	**93**	**69**	0.43 [0.26; 0.7]	**0.00062**
**IO-RT interval**	**RT >d14 after IO**	**94**	**69**	2.13 [1.24; 3.66]	**3.90E-03**
RT n of fractions	>3	94	69	0.55 [0.25; 1.2]	0.11
RT dose per fraction	>8	94	69	2.83 [1.74; 4.62]	5.40E-05
RT total dose EQD2	>36	94	69	1.79 [1.11; 2.9]	0.017
N of organs involved	**>or= 3**	94	69	1.23 [0.77; 1.99]	0.39
Involved: Node	Yes	94	69	0.98 [0.58; 1.65]	0.94
Involved: Brain	Yes	94	69	1.75 [0.7; 4.41]	0.27
Involved: Liver	Yes	94	69	2.65 [1.63; 4.33]	0.00019
Involved: Head&neck	Yes	94	69	2.59 [0.63; 10.7]	0.25
Involved: Bone	Yes	94	69	1.1 [0.53; 2.31]	0.80
Involved: Sub cutaneous	Yes	94	69	0.79 [0.46; 1.35]	0.38
Involved: Other	Yes	94	69	0.93 [0.55; 1.57]	0.79
Involved: Lung	Yes	94	69	1.28 [0.79; 2.06]	0.31
Involved: Adrenal gland	Yes	94	69	1.03 [0.32; 3.27]	0.97
Irradiated: node	Yes	94	69	0.78 [0.47; 1.28]	0.31
Irradiated: Brain	Yes	94	69	1.81 [0.56; 5.8]	0.36
Irradiated: Liver	Yes	94	69	2.49 [1.32; 4.67]	0.010
Irradiated: Head&neck	Yes	94	69	11.12 [1.39; 88.89]	0.089
Irradiated: Bone	Yes	94	69	0.29 [0.04; 2.08]	0.13
Irradiated: Sub cutaneous	Yes	94	69	0.96 [0.51; 1.8]	0.91
Irradiated: Other	Yes	94	69	1.41 [0.34; 5.78]	0.65
Irradiated: Lung	Yes	94	69	0.75 [0.42; 1.34]	0.32
PD-L1 (tumor cells)	>5%	40	21	0.52 [0.21; 1.25]	0.14
PD-L1 (immune cells)	>5%	39	21	0.57 [0.23; 1.42]	0.21
Absolute neutrophil count	>7.5 G/l	85	63	1.2 [0.66; 2.18]	0.57
Absolute lymphocyte count	<1 G/l	85	63	1.31 [0.74; 2.32]	0.36
**LDH**	**>250**	**86**	**66**	2.15 [1.3; 3.55]	**0.0041**
Albumin	<35	64	51	2.03 [0.86; 4.83]	0.14
**CRP**	**>10 mg/l**	**90**	**66**	1.77 [1.07; 2.92]	**0.028**
NLR	>6	85	63	1.61 [0.9; 2.85]	0.12
**RMH**	RMH > 0	58	48	2.31 [1.25; 4.28]	0.0055
**GRim**	High	55	45	3.29 [1.74; 6.21]	0.00018
**RS CD8 T cells: entropy across all lesions**	**>1.09**	**87**	**65**	1.67 [1.02; 2.75]	**0.040**
RS CD8 T cells: minimal	>1.61	91	68	0.54 [0.34; 0.88]	0.014
value of non-irradiated lesions	High (>Q3)	46	33	3.27 [1.58; 6.77]	0.0011

IO: immuno-oncology; RT: radiotherapy; EQD2: equivalent dose in 2-Gy fraction; LDH: lactate dehydrogenase; CRP: C-reactive protein; NLR: neutrophil to lymphocyte ratio; RMH: Royal Marsden Hospital score; Grim: Gustave Roussy immune-score; RS: radiomics score.

Table S4. Multivariate analysis of OS and PFS

OS	Variable tested	Hazard Ratio [95% CI]	p
Performance status	PS 1-2	1.57 [0.72; 3.45]	0.26
IO-RT interval	>d14	2.38 [0.68; 8.37]	0.18
LDH	>250	2.07 [0.99; 4.31]	0.052
CRP	>10 mg/l	3.34 [1.48; 7.52]	0.0036
RS CD8 T cells: entropy across all lesions	>1.09	2.64 [1.21; 5.75]	0.015

PFS	Variable tested	Hazard Ratio [95% CI]	p
Performance status	PS 1-2	4.6 [2.28; 9.28]	0.000021
IO-RT interval	>d14	1.82 [0.86; 3.85]	0.12
LDH	>250	1.82 [0.99; 3.34]	0.054
CRP	>10 mg/l	1.16 [0.61; 2.21]	0.65
RS CD8 T cells: entropy across all lesions	>1.09	1.23 [0.68; 2.21]	0.49

IO: immuno-oncology; RT: radiotherapy; LDH: Lactate dehydrogenase; CRP: C-reactive protein; NLR: neutrophil to lymphocyte ratio; RS: radiomics score.

Table S5. Multivariate analysis of OS and PFS including the centre of inclusion.

OS	Variable tested	Hazard Ratio [95% CI]	p
Performance status	PS 1-2	1.70 [0.76; 3.81]	0.20
IO-RT interval	>d14	2.86 [0.77; 10.60]	0.12
LDH	>250	2.11 [1.03; 4.33]	0.004
CRP	>10 mg/l	3.54 [1.55; 8.06]	0.0026
RS CD8 T cells: entropy across all lesions	>1.09	2.59 [1.19; 5.62]	0.016
Centre:Erlangen	Ref :Gustave Russy	NC	NC
Centre:Ghent		1.42 [0.64; 3.16]	0.38

PFS	Variable tested	Hazard Ratio [95% CI]	p
Performance status	PS 1-2	4.59 [2.27; 9.31]	0.000023
IO-RT interval	>d14	1.81 [0.77; 4.25]	0.18
LDH	>250	1.82 [0.99; 3.35]	0.054
CRP	>10 mg/l	1.16 [0.60; 2.24]	0.67
RS CD8 T cells: entropy across all lesions	>1.09	1.23 [0.68; 2.22]	0.49
Centre:Erlangen	Ref :Gustave Russy	NC	NC
Centre:Ghent		0.99 [0.51; 1.94]	0.98

IO: immuno-oncology; RT: radiotherapy; LDH: Lactate dehydrogenase; CRP: C-reactive protein; NLR: neutrophil to lymphocyte ratio; RS: radiomics score. NC: not computable

Supplementary Figures

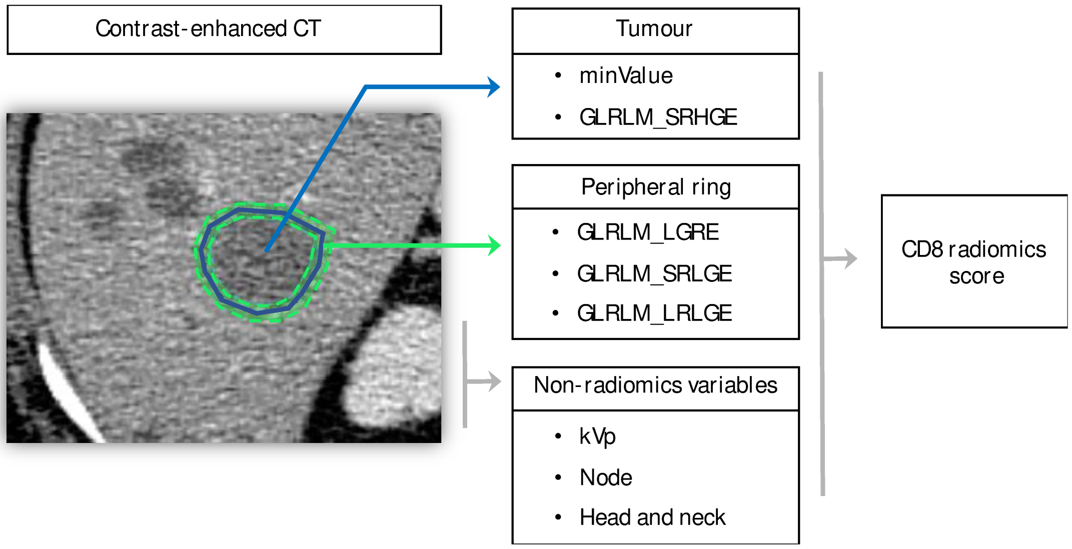

Figure S1. Description of the volumes of interest used to compute the radiomic score. Two volumes of interest (VOIs) were segmented for each lesion: (1) the lesion itself and (2) a peripheral ring (2mm on both sides of the tumor). The CD8 radiomics score integrated two radiomics features from the tumour, three radiomics features from the peripheral ring, and two non-radiomics features: the kilovoltage peak (kVp) and the location of the volume (node, or head and neck).
minValue: minimal value; GLRLM: Gray-Level Run Length Matrix; SRHGE: short-run high gray-level emphasis; LGRE: low gray-level run emphasis; SRLGE: short-run low gray-level emphasis; LRLGE: long-run low gray-level emphasis.

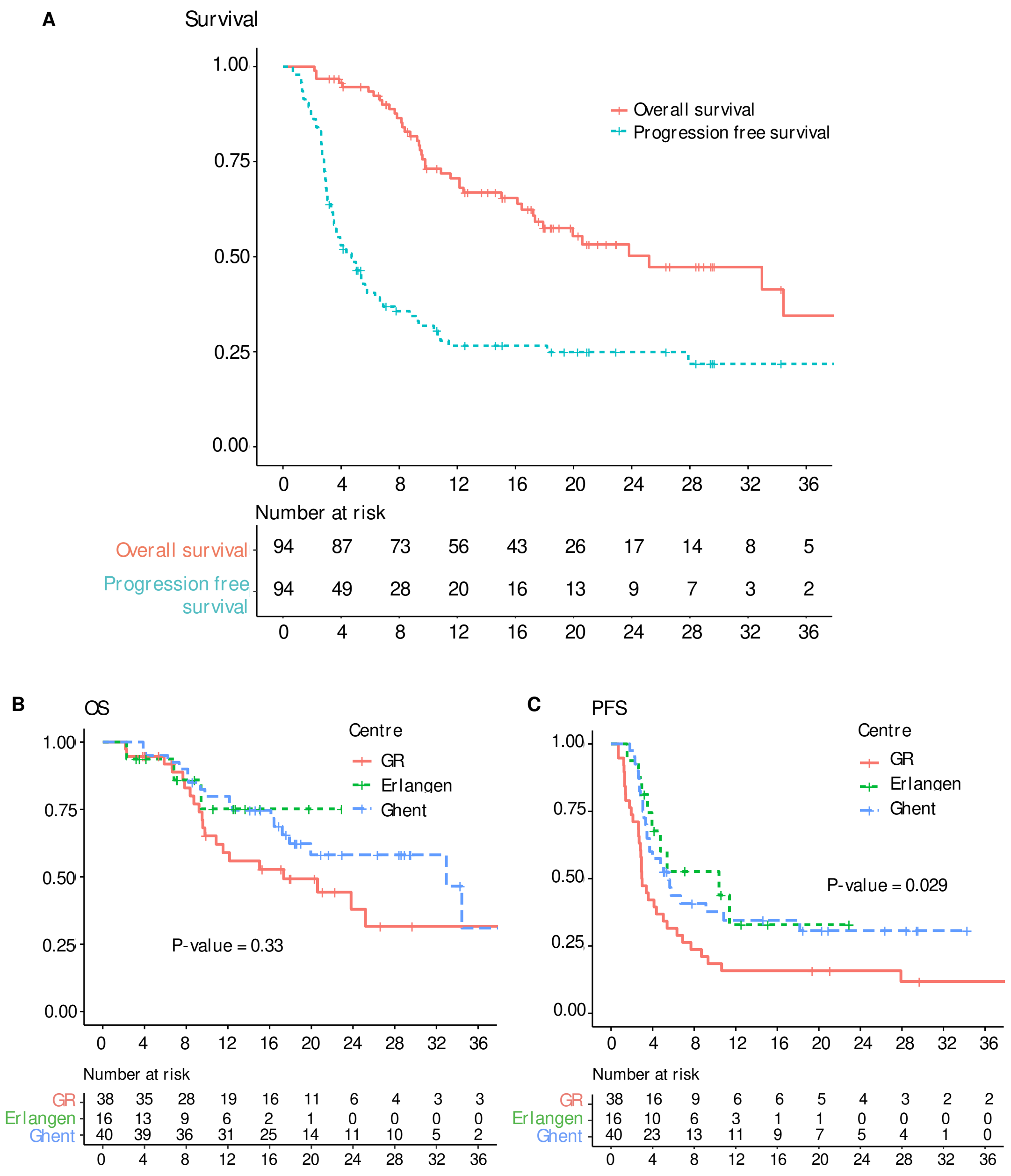

Figure S2. Kaplan-Meier curves of overall and progression-free survival of the whole cohort (A). B and C: Overall survival and progression-free survival curves by centre.

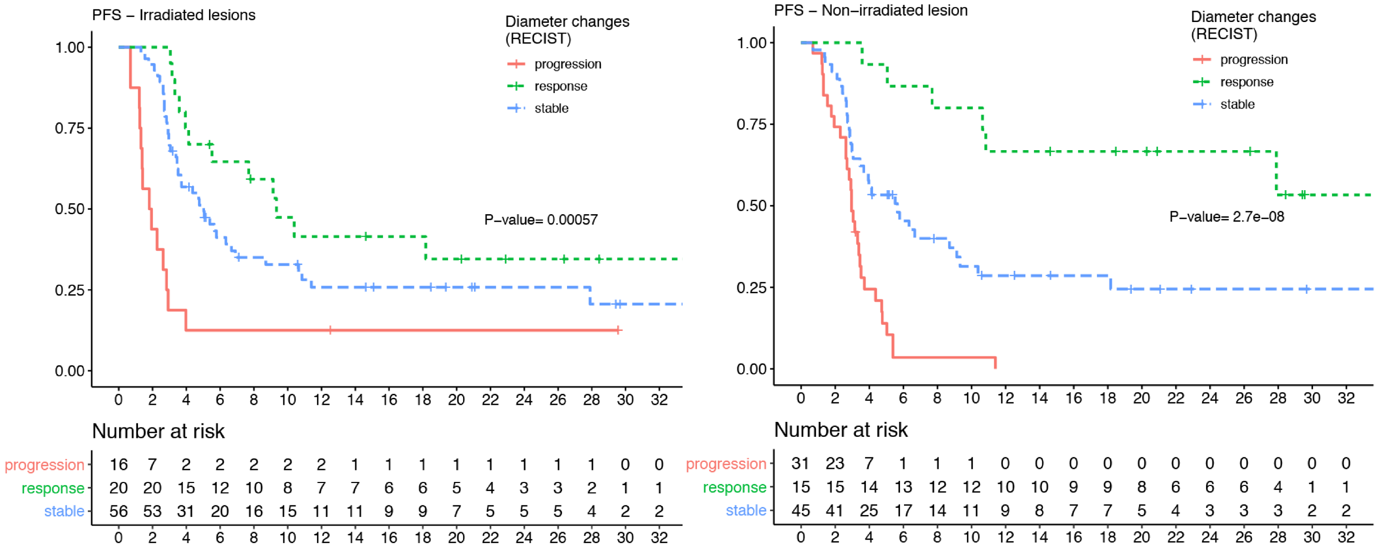

Figure S3. Kaplan-Meier curves of progression-free survival according to aggregate diameter changes of irradiated lesions (left) or non-irradiated lesions (right)

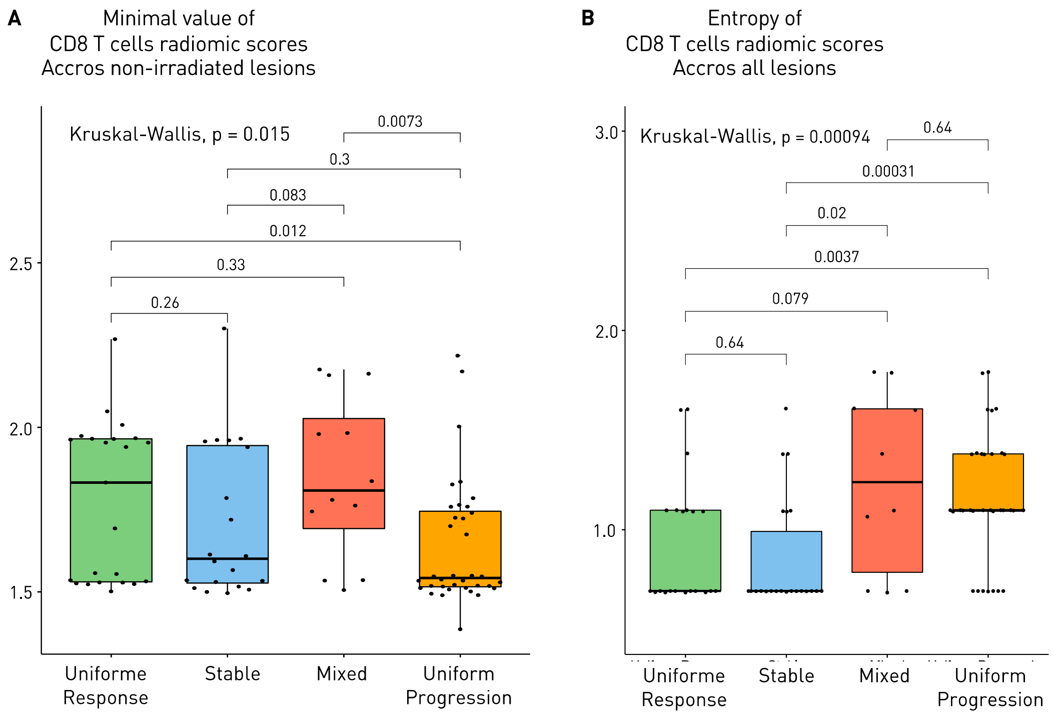

FIGURE S4. Association between patterns of response and CD8 T-cells score. A: minimal value of non-irradiated lesions, B: and entropy across all the different lesions.

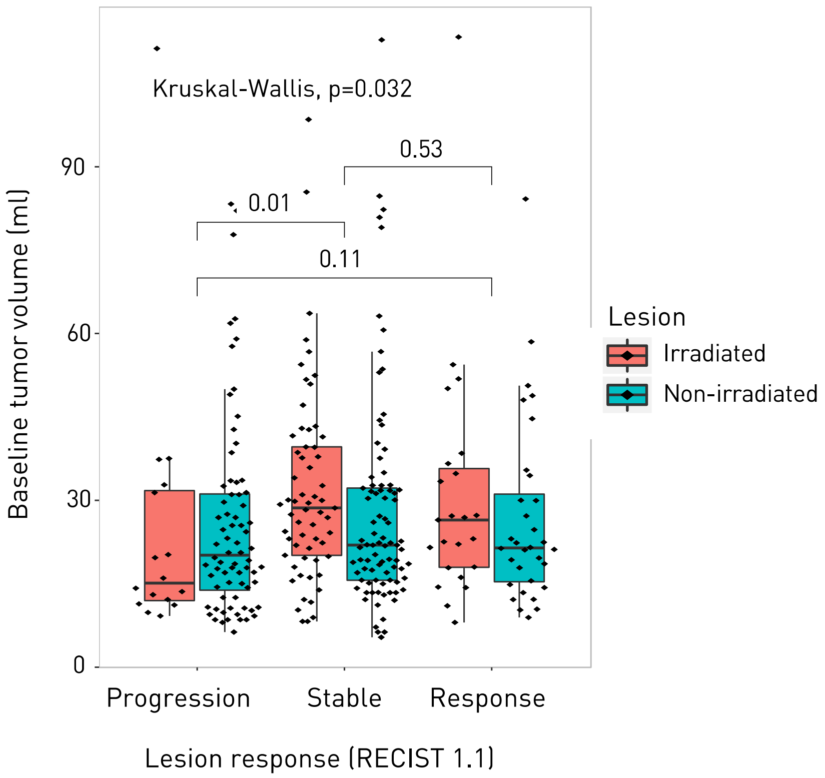

Figure S5. Lesion response according to tumour size. Responding lesions corresponded to lesions with a decrease in tumor size > 30% (partial response and complete response).
